# Serum anti-flagellin and anti-lipopolysaccharide immunoglobulins as predictors of linear growth faltering in Pakistani infants at risk for environmental enteric dysfunction

**DOI:** 10.1371/journal.pone.0193768

**Published:** 2018-03-06

**Authors:** Sana Syed, Najeeha T. Iqbal, Kamran Sadiq, Jennie Z. Ma, Tauseef Akhund, Wenjun Xin, Sean R. Moore, Enju Liu, Shahida Qureshi, Kerri Gosselin, Andrew Gewirtz, Christopher P. Duggan, S. Asad Ali

**Affiliations:** 1 Department of Pediatrics, University of Virginia, Charlottesville, VA, United States of America; 2 Department of Pediatrics, the Aga Khan University, Karachi, Pakistan; 3 Division of Gastroenterology, Hepatology and Nutrition, Boston Children’s Hospital, Boston, MA, United States of America; 4 Department of Public Health Sciences, University of Virginia, Charlottesville, VA, United States of America; 5 Center for Inflammation Immunity & Infection, Georgia State University, Atlanta, GA, United States of America; 6 Departments of Global Health and Population, Harvard T.H. Chan School of Public Health, Boston, MA, United States of America; 7 Nutrition, Harvard T.H. Chan School of Public Health, Boston, MA, United States of America; New York State Department of Health, UNITED STATES

## Abstract

**Background:**

Environmental Enteric Dysfunction (EED) in children from low-income countries has been linked to linear growth declines. There is a critical need to identify sensitive and early EED biomarkers.

**Objective:**

Determine whether levels of antibodies against bacterial components flagellin (flic) and lipopolysaccharide (LPS) predict poor growth.

**Design/Methods:**

In a prospective birth cohort of 380 children in rural Pakistan blood and stool samples were obtained at ages 6 and 9 months. Linear mixed effects models were used to examine longitudinal associations between quartiles of anti-flic and anti-LPS antibodies and changes in LAZ, WAZ and WLZ scores. Spearman’s correlations were measured between anti-flic and anti-LPS immunoglobulins with measures of systemic/enteric inflammation and intestinal regeneration.

**Results:**

Anti-LPS IgA correlated significantly with CRP, AGP and Reg1 serum at 6mo and with MPO at 9mo. In multivariate analysis at 6mo of age, higher anti-LPS IgA levels predicted greater declines in LAZ scores over subsequent 18mo (comparing highest to lowest quartile, β (SE) change in LAZ score/year = -0.313 (0.125), p-value = 0.013). Anti-flic Ig A in the two highest quartiles measured at 9mo of age had declines in LAZ of -0.269 (0.126), p = 0.033; and -0.306 (0.129), p = 0.018 respectively, during the subsequent 18mo of life, compared to those in the lowest quartile of anti-flic IgA.

**Conclusions and relevance:**

Elevated anti-flic IgA and anti-LPS IgA antibodies at 6 and 9mo, predict declines in linear growth. Systemic and enteric inflammation correlated with anti-LPS IgA provides mechanistic considerations for potential future interventions.

## Introduction

Approximately 3 million children under the age of 5 years die annually due to malnutrition[1], and each year about 525,000 children under 5 die due to diarrhea globally[2]. Evidence has arisen from clinical and animal model studies of undernutrition and diarrhea to support a reciprocal, additive relationship of recurrent enteric infections, along with subclinical infections and malnutrition with resultant environmental enteric dysfunction (EED). EED is a subclinical condition of the small intestine characterized by villous atrophy, crypt hyperplasia, increased intestinal permeability, inflammatory cell infiltration, and malabsorption [3–5]. EED is hypothesized to be mediated by aberrant innate and adaptive host immune responses in the context of chronic mucosal inflammation directed at a normal or possibly abnormal enteric microbiome [4,6].

In other chronic enteric inflammatory disease states such as inflammatory bowel disease (IBD), bacterial flagellin has been proposed to be involved in triggering an altered adaptive response by the mucosal immune system. Flagellin is the monomeric subunit that comprises bacterial flagella and is therefore expressed abundantly by motile gut bacteria [7]. The presence of flagellin on the luminal side of intestinal epithelial cells does not trigger inflammation; however, flagellin binds Toll-like receptor 5 (TLR5) found predominantly on the basolateral side of these cells, triggering an intense inflammatory response [8, 9, 10, 11]. This may occur during situations when the intestinal cell or mucosal barrier is breached, either by 1) translocation of flagellin across the epithelial cell monolayer as occurs with the pathogen Salmonella enterica serovar Typhimurium [9] or 2) mucosal injury that exposes the intestinal basolateral membrane to the hostile environment of the gut luminal contents. The resulting inflammatory response forms a major line of innate immune defense against bacterial infection, but such a response could also be a contributory factor to the pathogenesis of EED. Thus the goal of this work was to determine if flagellin-specific (flic) immunoglobulins, which would indicate that sufficient flagellin had penetrated the gut and triggered the innate immune system, are associated with growth faltering due to EED. Flagellin monomers purified from a human commensal Escherichia coli strain (F-18) serve as a “generic” flagellin, antibodies to which cannot distinguish between bacterial species and serotypes [12,13]. Levels of IgG and IgA that recognized bacterial lipopolysaccharide (LPS) were also measured because some studies have observed these immunoglobulins to be elevated in IBD [14].

Therefore, the aims of this paper were to: 1) Determine whether levels of flic- and LPS-specific IgA and IgG were associated with changes in growth as measured by Z scores of weight-for-height (WLZ), weight-for-age (WAZ), and height-for-age (LAZ). 2) Explore the associations of flic- and LPS-specific IgA and IgG at 6 and 9 months with biomarkers of systemic/enteric inflammation and intestinal regeneration (C-reactive protein (CRP), alpha- 1-acid glycoprotein (AGP), ferritin, myeloperoxidase (MPO), neopterin (NEO), regenerating gene 1β (Reg1b) stool and serum) in a cohort of infants at risk for growth faltering. We hypothesized that higher levels of a mucosal inflammatory response (i.e. as assessed by plasma flic- and LPS-specific IgA and IgG) would be associated with decreased LAZ, WAZ, and WLZ scores.

## Methods

### Study design and participants

Subjects included in this analysis were part of a prospective community-based active surveillance birth cohort with an intervention arm that was designed to investigate various biomarkers of growth faltering, response to Ready-to-Use-Therapeutic-Food (RUTF) and endoscopic evaluation of children with an inadequate growth response to RUTF **(S1 Fig)** [15,16]. The study site was in the rural district of Matiari, located in the province of Sindh about 200 km (124 miles) north of Karachi, Pakistan. The Department of Paediatrics and Child Health at Aga Khan University (AKU), Pakistan has established research infrastructure and relationships in this area in collaboration with the Government of Pakistan since 2002 for the purpose of community-based research.

Enrollment and assessment of newborns was done during routine surveillance of pregnant women of reproductive age (13–49 years) by community health workers. At the time of the first study visit, mothers were asked to approximate the duration of pregnancy and this was used to calculate birth gestational age of the infants. We have added this as a recall bias to our limitation. Study inclusion criteria were: 1) newborns aged up to 14 days; 2) absence of any major congenital abnormalities and; 3) ability to obtain informed consent from parents or guardians. Infants of families planning to move out of the study area within 6 months of birth were excluded from the trial. Enrolled children were followed from birth (0 to 14 days) until 18 months of age with weekly home visits during the study period from October 2012 to November 2015. All families enrolled in the study were provided with cell phone contact information of key study physicians to enable direct and immediate contact in the case of any urgent medical need by the study participants. Monthly measurements were recorded by trained community health workers using standard techniques: child’s weight using a digital infant balance with 20-g precision (TANITA 1584) and measured the child’s length with 1 mm precision (using a rigid length board with a movable foot piece). Standardization of measurements was ensured through regular staff training and cross checks.

For purposes of comparison, anti-flic and anti-LPS antibodies were also measured in a cohort of 36 healthy infants seen at Boston Children’s Hospital. After institutional review board approval was obtained, any infant from birth to 12 months of age with an excess blood sample in the Boston Children’s Hospital central laboratory was eligible for inclusion. Subjects were excluded from participation in the research study if 1) their LAZ was <-2 or >2; 2) their WAZ was <-2 or >2; 3) no growth data were available for the review and analysis; 4) there was evidence of a recent fever (temperature >38.5 C in the past 48 h); or 5) there was any evidence of a systemic illness (e.g., malignancy). A total of 67 infants were screened for enrollment, and 31 infants were excluded. The mean age of the remaining 36 infants at the time of the blood draw was 9.5 months (range: 5–12 months).

### Biological specimen collection and measurement of biomarkers

Blood was obtained from enrolled children in Pakistan at 6 and 9 months of age. **S1 Table** summarizes the rationale and purpose behind their measurements in our study.

Blood samples were centrifuged and plasma removed within 2 h of blood collection in the field site research laboratory. Samples were transported at 4°C from Matiari to the AKU Infectious Disease Research Lab (IDRL) under cold chain maintenance. Aliquots were stored in -80°C freezers with an automatic backup electricity generator. Peripheral blood samples taken at 6 and 9 months were tested for flic- and LPS-specific IgA and IgG concentrations measured by ELISA, as previously reported [17]. Microtiter plates were coated with purified E. coli flagellin (100ng/well) or purified E. coli LPS (2 μg/well). Serum samples from study participants were diluted 1:200 and applied to wells coated with flagellin or LPS. After incubation and washing, the wells were incubated with anti-human IgA (KPL) or IgG (GE Healthcare) coupled to horseradish peroxidase. Quantification of total immunoglobulins was performed using the colorimetric peroxidase substrate tetramethylbenzidine, and optical density (OD) was read at 450 nm with an ELISA plate reader. Data are reported as OD corrected by subtracting background levels, which were determined by readings in samples lacking serum. Commercial ELISA kits for the estimation of regenerating gene 1β (Reg1) (TechLab, Blacksburg, Virginia) in stool and serum. For intestinal inflammation, commercial ELISA kits for the estimation of myeloperoxidase (MPO) (Immunodiagnostic AG, Stubenwald-Allee, Bensheim) and neopterin (NEO) (GenWay Biotech, San Diego, CA) in stool samples were used as reported previously [18]. Biomarkers of systemic inflammation and C-reactive protein (CRP) and alpha- 1-acid glycoprotein (AGP) and ferritin were analyzed on Hitachi 902 analyzer (Roche Diagnostics, Holliston, MA). All protocols were followed as per manufacturers’ instructions. The final dilution for serum and stool biomarkers was determined by selecting the most appropriate concentration of a biomarker falling in the linear range of standard curve. Reg1B was performed in two dilutions of 1:40,000 and 1:100,000, NEO at the dilution of 1:250 and MPO at 1:500. All plates were read on Biorad iMark (Hercules, CA) plate reader.

### Statistical analysis

We used the WHO Child Growth Standards (WHO Anthro, Geneva, Switzerland) [19] to calculate z-scores, and assessed growth both as continuous measures of length-for-age z-score (LAZ), weight-for-age z-score (WAZ) and weight-for-length z-score (WLZ); and as categorized variables of stunting as LAZ < −2 SD (standard deviation), underweight as WAZ < −2 SD and wasting as WLZ < −2 SD. In accordance with WHO recommendations, we set all extreme LAZ (<-6 or >6), WLZ (<-5 or >5), and WAZ (<-6 or >5) values to missing as recommended [20]. Participant descriptive statistics were presented as means (standard error, SE) and as percentage (95% confidence interval, CI) for continuous and categorical outcomes. Due to the skewed distributions of most biomarkers, Spearman correlations were estimated between flic- and LPS-specific IgA and IgA at 6 and 9 months with biomarkers of systemic and enteric inflammation and intestinal regeneration (CRP, AGP, ferritin, MPO, NEO, Reg1b stool and serum) along with associated 95% confidence intervals.

Monthly Z-scores (LAZ/WAZ/WLZ) from birth to 18 months were modeled as the longitudinal growth responses in a linear mixed effects model using the following equation for the i^th^ child at the j^th^ time t_ij_ with the biomarker value (biomarker_i_):

$$LAZ_{ij}=\left(\beta_{0}+\alpha_{0i}\right)+\beta_{1}\;biomarker_{i}+\left(\beta_{2}+\alpha_{2i}\right)\;t_{ij}+\beta_{3}\;biomarker_{i}\;*\;t_{ij}+\varepsilon_{ij},$$

where βs are the fixed effects, αs are the random effects, and ε is the error term. Random intercept and random slope were specified in the model to allow subject-specific variation in the growth. An individual biomarker was considered as a fixed effect, and its association with the growth was characterized by its interaction with time. That is, the biomarker effect on the growth was quantified by its influence on the growth slope, the rate of growth change per year.

Due to their skewed distributions, biomarkers were divided into four quartiles as q1 (0-25th percentile), q2 (25th -50th percentile), q3 (50th- 75th percentile) and q4 (75th -100th percentile) at 6 and 9 months, and were considered categorical predictors. The lowest quartile (q1) was used as the reference group. To assess the association between each biomarker and the anthropometric outcomes, we first performed a series of univariate analyses, and then followed by multivariable analyses that adjusted for potential confounders including child sex, preterm birth, maternal age, maternal literacy, antibiotic use at baseline (6 or 9 months of age) and whether RUTF was received or not. Additional models were also computed using all four biomarkers as continuous variables instead of quartiled predictors. These covariates were selected based on their P value <0.10 in univariate analysis or traditionally considered as a risk factor for the child growth outcomes in literature, or biological pathway plausibility informed by a hypothesized conceptual causal diagram **(S2 Fig)** for determinants of EED.

Growth was also modeled as time-to-event outcomes in Cox proportional hazards models to estimate hazard ratios (HRs) and corresponding 95% confidence intervals (CIs) for stunting, wasting and underweight across the quartiles of the each biomarker. Each growth outcome was modeled separately and the first time the child reached a score of < -2 SD signified an “event”. Children who did not develop the “event” were censored at the time of last anthropometric assessment. By design in our birth cohort, we had included children who were stunted/wasted or underweight at birth for this analysis but excluded those who already experienced the event of interest at baseline. E.g., for our stunting analysis we excluded children who were already stunted between age 0 to 6 months, with similar exclusions made for each of our wasting and underweight analysis. Similar to the linear mixed effects modeling, we first performed a series of univariate Cox regression and then multivariable analyses adjusting the same set of covariates. P-value for trend was calculated by including the median value within each quartile as a continuous term in the regression model. All analyses were performed using SAS version 9.4 (SAS Institute, Cary, NC, USA). Descriptive figures were made using GraphPad Prism version 6.00 for Windows and GraphPad Software (La Jolla California USA, www.graphpad.com).

Ethics Statement and Study Data. Institutional approval was granted by the Aga Khan University Ethical Review Committee, Harvard T.H. Chan School of Public Health Human Subjects Committee, the Boston Children’s Hospital Institutional Review board, and the University of Virginia Institutional Review Board. All parents provided written informed consent.

## Results

Summary statistics of variables from the cohort which were included in the multivariable linear mixed effects models at 6 and 9 months are shown in **Table 1**.

**Table 1:** Infant and Maternal Characteristics at six and nine months*.

	N (%) or Median (q1,q3)	
Maternal Baseline Characteristics	6 months	9 months
Maternal Literacy, no	310 (87.3%)	273 (87.5%)
Maternal Age, <30 y	158 (44.3%)	135 (43.1%)
Antenatal care received, yes	249 (68.0%)	209 (66.6%)
Breastfeeding, yes	361 (99.2%)	305 (97.4%)
Child Baseline Characteristics		
Male	184 (51.4%)	159 (50.8%)
Low birth weight, <2500g	129 (35.3%)	111 (35.4%)
Born preterm, <37 weeks	232 (64.8%)	201 (64.2%)
Length-for-age Z-score	-2.0 (-2.9, -1.3)	-2.2 (-2.8, -1.4)
Weight-for-length Z-score	-0.7 (-1.4, 0.2)	-0.8 (-1.6, 0.2)
Weight-for-age Z-score	-1.9 (-2.8, -1.1)	-1.9 (-2.8, -1.1)
Antibiotic use at the time of biomarker measurement, yes	41 (11.6%)	38 (12.4%)

Note: n = 376 at six month and n = 322 at nine month

*Summary statistics presented of variables included in the multivariable linear mixed effects models at six and nine months.

**Fig 1:**
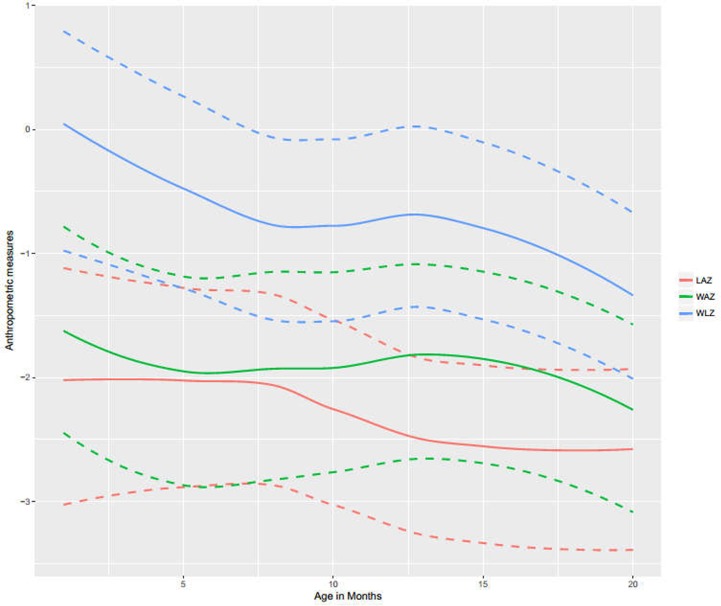
Median LAZ, WLZ and WAZ scores in Pakistani infants over 18 months of follow-up. Dashed lines show q1 and q3 quartiles. Abbreviations: LAZ = Length-for-Age Z score, WLZ = Weight-for-Length Z score and WAZ = Weight-for-Age Z score.

Maternal demographics were notable for high illiteracy (87% at both 6 and 9 months). Almost all infants were still breastfeeding at both time points (>97% at both 6 and 9 months). Cohort characteristics were notable for having equal gender distribution among infants, more than half born preterm (<37 weeks) and about one third having low birth weight. Median (q1, q3) LAZ, WAZ, and WLZ scores (**Fig 1**) trended downwards over 18 months of follow-up.

**Fig 2:**
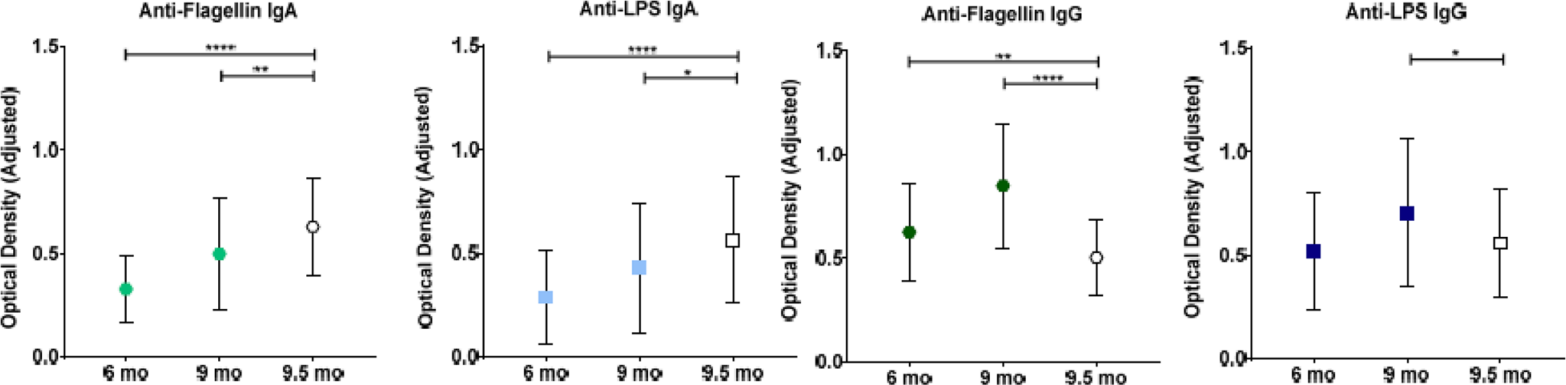
Anti-Flagellin and anti-LPS immunoglobulin concentrations in Pakistani infants. Closed shapes indicate means±SDs in Pakistani infants over the follow-up period. Open shapes indicate means±SDs values in 36 healthy Boston infants with a mean age of 9.5 months. n = 380 for all immunoglobulins in Pakistani infants at 6 and 9 months of age; n = 36 for all immunoglobulins in Boston infants at 9.5 months of age. **** p< 0.0001, ** p< 0.01 and * p< 0.05 for comparisons of mean biomarker concentrations in Pakistani infants with those of healthy Boston infants (unpaired t tests).

**Fig 2** illustrates the progressive increase in the concentrations of all four immunoglobulins over the follow-up period. Mean concentrations of both anti-flic IgA and anti-LPS IgA were significantly higher in the healthy Boston children than in the Pakistani children at both 6 and 9 months of age, whereas mean concentrations of anti-flic IgG were significantly higher in the Pakistani infants compared with healthy controls at both 6 and 9 months of age. Only anti-LPS IgG at 9 months of age was significantly higher in the Pakistani infants.

The interrelationships of all four **immunoglobulins** of bacterial translocation both with measures of systemic/enteric inflammation and intestinal regeneration (CRP, AGP, ferritin, MPO, NEO, Reg1b in stool and serum) and within themselves were measured using the Spearman correlation coefficients as summarized in **Table 2 and S2 Table**.

**Table 2:** Correlation coefficient matrix of immunoglobulins to flagellin and lipolysaccharide with measures of systemic inflammation and enteric inflammation & regeneration.

	At 6 months							At 9 months						
	Systemic inflammation			Enteric inflammation & regeneration				Systemic inflammation			Enteric inflammation & regeneration			
	CRP mg/L n = 320	AGP mg/dL n = 307	Ferritin ng/mL n = 320	MPO ng/mL n = 322	NEO nmol/L n = 322	Reg1b stool μg/g n = 319	Reg1b serum pg/mL n = 315	CRP mg/L n = 369	AGP mg/dL n = 308	Ferritin ng/mL n = 369	MPO ng/mL n = 377	NEO nmol/L n = 373	Reg1 stool μg/g n = 316	Reg1 serum pg/mL n = 316
At 6 months								At 9 months						
Anti-Flic IgA, OD	0.02	0.11	0.02	-0.03	0.06	0.002	0.21**	0.01	0.15*	0.04	0.07	-0.10	0.04	0.04
Anti-Flic IgG, OD	0.04	0.06	0.02	0.01	0.02	0.02	0.10	0.01	0.07	0.16*	0.07	-0.18**	0.11	0.05
Anti-LPS IgA, OD	0.12*	0.18*	0.06	-0.04	-0.03	0.04	0.20**	0.05	0.11	0.03	0.11*	-0.10	0.07	0.02
Anti-LPS IgG, OD	0.10	0.12*	0.03	-0.02	-0.03	-0.03	0.08	0.02	-0.005	0.10	0.03	-0.17*	0.10	-0.04

Note: Values are Spearman’s rank-order correlation coefficient.

***P<0.0001

**P<0.001

*P-value<0.05.

Abbreviations: Flagellin = Flic; Immunoglobulin = Ig; Lipopolysacccharide = LPS; alpha glycoprotein = AGP; C-reactive protein = CRP; Myeloperoxidase = MPO; Neopterin = NEO; regenerating gene 1β = REG1b

We found that at 6 months anti-LPS IgA and IgG were significantly correlated with measures of systemic inflammation (r = 0.12, 0.18 for CRP and AGP; ρ_s_ = 0.12 for AGP respectively). At 9 months anti-LPS IgA was significantly correlated with enteric inflammation (ρ_s_ = 0.11 with MPO) but interestingly, there was significant negative correlation of anti-LPS IgG with NEO, ρ_s_ = -0.17. Anti-flic IgA and IgG significantly correlated with systemic inflammation (ρ_s_ = 0.15 with AGP and ρ_s_ = 0.16 with ferritin respectively) at 9 months with anti-flic IgG also correlating significantly with enteric inflammation (NEO, ρ_s_ = -0.18); this too was a negative relationship, however. Anti-Flic IgA was highly correlated with the other three immunoglobulins at 6 and 9 months, ρ_s_ = 0.57, 0.63 and 0.40 at 6 months and ρ_s_ = 0.56, 0.82, and 0.31 at 9 months with anti-Flic IgG, anti-LPS IgA, and anti-LPS IgG, respectively; p<0.001. With the exception of anti-Flic IgG, the remaining three immunoglobulins at 6 months were all significantly correlated with their 9 month levels.

In the linear mixed effects models **(Table 3, and S3 and S4 Tables)**, we analyzed the association of each of our four immunoglobulins with the longitudinal anthropometric data. At 6 months of age, the highest quartile of anti-LPS IgA was associated with β (SE) change in LAZ of -0.33 (0.12) per year, compared to the lowest quartile of anti-LPS IgA (<0.17 OD) (p-value = 0.008). At 9 months of age, the two highest quartiles of anti-flic IgA were associated with β (SE) change per year in LAZ of -0.29 (0.13), p-value = 0.02; and -0.32 (0.13), p-value = 0.01 respectively, during the first 18 months of life, compared to the lowest quartile of anti-flic IgA. At 6 months of age, the second quartile of anti-LPS IgA was associated with β (SE) change per year in WLZ of 0.34 (0.15), p-value = 0.03, during the first 18 months of life, compared to the lowest quartile of anti-flic IgA. In our sub-analyses using the Cox proportional hazards models with immunoglobulins at 6 months of age **(S5 Table)**, those in the second (HR 1.57, 95%CI: 0.81, 3.05) and third quartile (HR 2.23, 95%CI: 1.15, 4.33) had an increased risk of stunting, however this increased risk was not sustained in the highest quartile (HR 0.96, 95%CI: 0.48, 1.91, P-trend = 0.89) when compared to children in first quartile of anti-LPS IgA level. Lastly, in multivariable linear mixed effects models **(S6 Table)** we analyzed the association of each of our four biomarkers of EED as continuous variables with the longitudinal anthropometric data. The only significant result of note was at 9 months of age, anti-Flic IgG was associated with β (SE) change in LAZ of -0.30 (0.15) per year (p-value = 0.05).

**Table 3:** The association of anti-flagellin and anti-lipopolysaccharide immunoglobulin concentrations at 6 and 9 months with annual Z score changes for length using linear mixed effects models.

Immunoglobulins at 6 months with annual ΔLAZ as outcome					Immunoglobulins at 9 months with annual ΔLAZ as outcome				
	unadj β (SE)	*p-value*	adj β (SE)^1^	*p-value*		unadj β (SE)	*p-value*	adj β (SE)^1^	*p-value*
**Flic IgA, OD**					**Flic IgA, OD**				
**Q1** <0.21	ref	—	ref	—	**Q1** <0.32	ref	—	ref	—
**Q2** 0.21 to <0.30	0.29(0.12)	0.02	0.21(0.12)	0.09	**Q2** 0.32 to <0.44	-0.22(0.13)	0.09	-0.22(0.13)	0.08
**Q3** 0.30 to <0.42	0.08(0.12)	0.52	0.05(0.12)	0.67	**Q3** 0.44 to <0.61	-0.25(0.13)	0.049	-0.29(0.13)	0.02
**Q4** >0.42	-0.05(0.12)	0.68	-0.04(0.12)	0.72	**Q4** >0.61	-0.30(0.13)	0.03	-0.32(0.13)	0.01
**Flic IgG, OD**					**Flic IgG, OD**				
**Q1** <0.47	Ref	—	Ref	—	**Q1** <0.65	Ref	—	Ref	—
**Q2** 0.47 to <0.60	0.03(0.13)	0.82	0.11(0.12)	0.39	**Q2** 0.65 to <0.82	-0.06(0.13)	0.63	-0.04(0.13)	0.73
**Q3** 0.60 to <0.75	0.10(0.12)	0.44	0.12(0.12)	0.33	**Q3** 0.82 to <1.03	-0.04(0.13)	0.78	-0.07(0.13)	0.59
**Q4** >0.75	-0.14(0.12)	0.26	-0.10(0.12)	0.41	**Q4** >1.03	-0.27(0.13)	0.04	-0.23(0.13)	0.07
**LPS IgA, OD**					**LPS IgA, OD**				
**Q1** <0.17	Ref	**—**	Ref	—	**Q1** <0.23	Ref	—	Ref	—
**Q2** 0.17 to <0.24	0.01(0.12)	0.91	-0.08(0.12)	0.51	**Q2** 0.23 to <0.36	-0.18(0.13)	0.16	-0.19(0.13)	0.14
**Q3** 0.24 to <0.34	-0.04(0.13)	0.78	-0.09(0.12)	0.47	**Q3** 0.36 to <0.56	-0.27(0.13)	0.037	-0.24(0.13)	0.07
**Q4** >0.34	-0.30(0.12)	0.02	-0.33(0.12)	0.008	**Q4** >0.56	-0.14(0.13)	0.28	-0.18(0.13)	0.16
**LPS IgG, OD**					**LPS IgG, OD**				
**Q1** <0.33	ref	—	ref	—	**Q1** <0.44	Ref	—	Ref	—
**Q2** 0.33 to <0.47	-0.07(0.12)	0.58	-0.04(0.13)	0.78	**Q2** 0.44 to <0.62	0.11(0.13)	0.38	0.16(0.13)	0.21
**Q3** 0.47 to <0.66	0.14(0.12)	0.25	0.15(0.12)	0.23	**Q3** 0.62 to <0.89	-0.05(0.13)	0.69	-0.05(0.13)	0.70
**Q4** >0.66	-0.10(0.12)	0.40	-0.08(0.12)	0.54	**Q4** >0.89	0.09(0.13)	0.48	0.06(0.13)	0.65

Note

^1^Adjusted for child sex (male/female), preterm birth (yes/no), maternal age (≥30, <30years), maternal literacy (yes/no), antibiotic use (yes/no), RUTF (yes/no). Abbreviations: Flic = Flagellin; LPS = Lipopolysaccharide; IgA = Immunoglobulin A; IgG = Immunoglobulin G; ΔLAZ = change in Length-for-age Z scores; RUTF = ready to use therapeutic food.

## Discussion

The present study reports associations of flic- and LPS- specific immunoglobulins, which are biomarkers of bacterial translocation, and growth faltering in 380 Pakistani children at risk of EED. Notable findings included: (1) while we observed a clear trend of increasing anti-LPS and anti-flic IgA and IgG concentrations over the first year of life, anti-flic and anti-LPS IgA were both lower than in our Boston controls; (2) anti-LPS IgA correlated significantly with serum CRP, AGP and Reg1 at 6 months and with fecal MPO at 9 months; (3) high anti-LPS IgA levels at 6 months of age and higher anti-flic IgA levels at 9 months of age were associated with a trend to greater decrease in LAZ scores over 18 months; (4) Compared to children in first quartile of anti-LPS IgA level, those in the second and third quartile had an increased risk of stunting.

A longitudinal study studying the growth of 590 Tanzanian infants and the relationship of these same flic- and LPS-specific immunoglobulins was recently published but with contrasting results [21]. While in both studies, anti-LPS and anti-flic IgA and IgG concentrations increased over the first year of life, Tanzanian children had significantly higher antibody (both IgA and IgG) concentrations compared with healthy Boston controls, whereas in the Pakistani cohort the IgA to Flic and LPS was lower at both 6 and 9 mo when compared to the Boston controls. In Tanzania, infants at 6 weeks of age in the highest quartile of all the EED antibody concentrations were approximately twice as likely to develop underweight (WAZ<-2) after adjustment for covariates (P-trend < 0.05) than were infants with Ig concentrations in the lowest quartile. Children with increased concentrations of anti-flic IgA were also more likely to become wasted (WHZ<-2); however, there was no association between any of the markers and subsequent stunting. In Pakistan, infants at 6 months of age with anti-LPS IgA in the highest quartile had significant decreases in vertical growth as measured by β (SE) change in LAZ of -0.313 (0.125) per year (p-value = 0.013). Using stunting as our outcome, we showed that children with higher anti-LPS IgA levels (in the second (HR 1.57, 95%CI: 0.81, 3.05) and third quartile (HR 2.23, 95%CI: 1.15, 4.33)) were approximately twice as likely to develop stunting, after adjustment for covariates, than were infants with Ig concentrations in the lowest quartile. Pakistani infants at 9 months of age with anti-flic IgA in the two highest quartiles of anti-flic IgA were similarly associated with significant decreases in vertical growth as indicated by β (SE) change per year in LAZ of -0.269 (0.126), p-value = 0.033; and -0.306 (0.129), p-value = 0.018 respectively, during the first 18 months of life, compared to the lowest quartile of anti-flic IgA. This was also true of infant with higher anti-LPS IgA levels (β (SE) change in LAZ of -0.255 (0.127) per year during the first 18 months of life, compared to the lowest quartile of anti-LPS IgA (p-value = 0.045)). Neither the Tanzanian study nor ours measured total IgA; therefore we are unable to determine if the difference in comparison with the same Boston controls was due to an underlying IgA deficiency in our Pakistani cohort. Given that IgA deficiency varies in prevalence depending on ethnic background [22], such an analysis would be worth conducting in future studies. Further, the study designs of the two projects were different. The Tanzanian study excluded subjects from participation in the research study if their LAZ was <-2 or > 2; therefore, the Tanzanian cohort was healthier at baseline by design.

Our results are supported by a recent case-control field study in Fortaleza, Brazil investigating biomarkers of EED, where Guerrant, et al. reported that anti-flic IgA and anti-LPS IgA (n = 292; r = 0.15, p = 0.011 and r = 0.14, p = 0.017) were significantly correlated with more severe stunting at the time of enrollment but were not predictive of subsequent growth (outcome defined as delta LAZ) [23]. This study however differed in design from ours in that, in the Brazilian study, malnourished or “case” children were initially defined as having WAZ scores <-2 and, to the extent possible, age and gender matched ‘non-malnourished controls’ were defined as having a WAZ greater than -1; biomarkers were then measured in these cases and controls. Follow up anthropometry at 2–6 months after initial sampling was also done to assess these biomarkers as predictors of subsequent growth. This differs from our study, in which monthly measures of growth were made over 18 months of follow-up with biomarkers measured at two time-points in the first year of life when presumably the effect of the environment would come into play. Despite these differences, our study and the field study conducted by Guerrant, et al. support the role of anti-flic and anti-LPS IgA as biomarkers of EED or predictors of subsequent stunting.

Using stool enteropathogen and biomarker data from within the framework of the Etiology, Risk Factors and Interactions of Enteric Infections and Malnutrition and the Consequences for Child Health and Development (MAL-ED) birth cohort study that included children from eight countries, Kosek et al. recently reported associations between enteropathogens and linear growth mediated through systemic inflammation[24]. At age group 4–11 months, fecal MPO was negatively associated with sample Z-scores of lactulose:mannitol (L:M) assays (assessing intestinal permeability) while in children aged 12–21 months MPO concentration was positively associated with AGP, while NEO was negatively associated with AGP. In contrast to this, we found early positive associations at six months of measures of intestinal permeability/ bacterial translocation (anti-LPS IgA and IgG) with CRP and AGP with anti-LPS IgA significantly and positively correlating with MPO. Similar to Kosek et al., we found significant negative correlation of our measures of intestinal permeability/ bacterial translocation (anti-LPS IgG) with NEO, ρ_s_ = -0.17. Of note, in a separate analysis studying the effect of biomarkers of systemic inflammation (CRP, Ferritin, AGP) and enteric inflammation (MPO, Neopterin) on growth, we found that high levels of ferritin, CRP and AGP were significantly associated with decreased annual LAZ scores [16]. In this parent analysis, we also found that MPO is tightly correlated with both CRP (both 6 and 9 months) and AGP (at 6 months only) with NEO not correlating with MPO at any time point and only with ferritin at 9 months.

There are several strengths of our study which merit mention. We followed a large cohort of children closely over an 18mo period of follow-up, a challenging feat in a population which was geographically spread out with poor routes of transportation. Our monthly repeated measures of growth (length and weight) along with our mixed methods modeling (using time varying variables) allowed our analysis to take into consideration that normal human growth is pulsatile with intervals of rapid growth which are separated by periods of no measurable growth [25]. We measured serum immunoglobulins at two time points in the first year of life (6 and 9 months of age) and we included multiple potentially confounding factors in our multivariable models. Lastly, we evaluated growth faltering as changes in linear slope of z scores for length/weight/weight for length while additionally categorizing growth into stunting, wasting and underweight. We were limited in that we used growth faltering during the first 18 months of life as a clinical substitute of EED and that we did not gather dietary intake data. Furthermore, the Boston control group was small, from a much broader range of ages, including much older children, all of which were grouped together rather than trying to perform any form of age-matching. We were also limited by maternal recall bias in how birth gestational age of the study infants was estimated. Ideally, we would have been able to confirm presence of EED using gold standards such as intestinal biopsies or L:M differential absorption ratios, but the scarcity of these data made such an analysis challenging.

In summary, serum levels of both anti-LPS and anti-flic IgA and IgG concentrations increased over the first year of life. Higher levels of both flic- and LPS-specific IgA were associated with linear faltering over the first 18 months of life. Children with higher anti-LPS IgA levels had an increased risk of stunting. Future directions could include the evaluation of serum IgA to stool ratios as a markers of alterations in the intestinal microbial communities and mucosal immunity in malnourished children. Further studies relating these biomarkers to EE small bowel histology would help shed further light on the alterations in the intestinal immune response.

## Supporting information

S1 Fig Flow of study Participants during 18 month study period. (TIF)

S2 Fig Hypothesized causal pathways for environmental enteric dysfunction (EED). (TIF)

S1 Table Summary of biomarkers to assess environmental enteric dysfunction from parent study. (DOCX)

S2 Table Correlation coefficient matrix of anti-flagellin and anti-lipopolysaccharide immunoglobulins. (DOCX)

S3 Table The Association of anti-flagellin and anti-lipopolysaccharide immunoglobulin concentrations at 6 and 9 months with annual Z score changes for weight using linear mixed effects models. (DOCX)

S4 Table The association of anti-flagellin and anti-lipopolysaccharide immunoglobulin concentrations at 6 and 9 months with annual Z score changes for weight-for-length using linear mixed effects models. (DOCX)

S5 Table The association of anti-flagellin and anti-lipopolysaccharide immunoglobulin concentrations at 6 months with subsequent stunting, wasting, and underweight. (DOCX)

S6 Table The association of continuous anti-flagellin and anti-lipopolysaccharide immunoglobulin concentrations at 6 and 9 months with annual Z score changes for length using linear mixed effects models. (DOCX)
