# Supplementary figures and images for: Serum anti-flagellin and anti-lipopolysaccharide immunoglobulins as predictors of linear growth faltering in Pakistani infants at risk for environmental enteric dysfunction
Source: PLoS One. 2018 Mar 6;13(3):e0193768. doi: 10.1371/journal.pone.0193768 (PMC5839587; doi:10.1371/journal.pone.0193768)

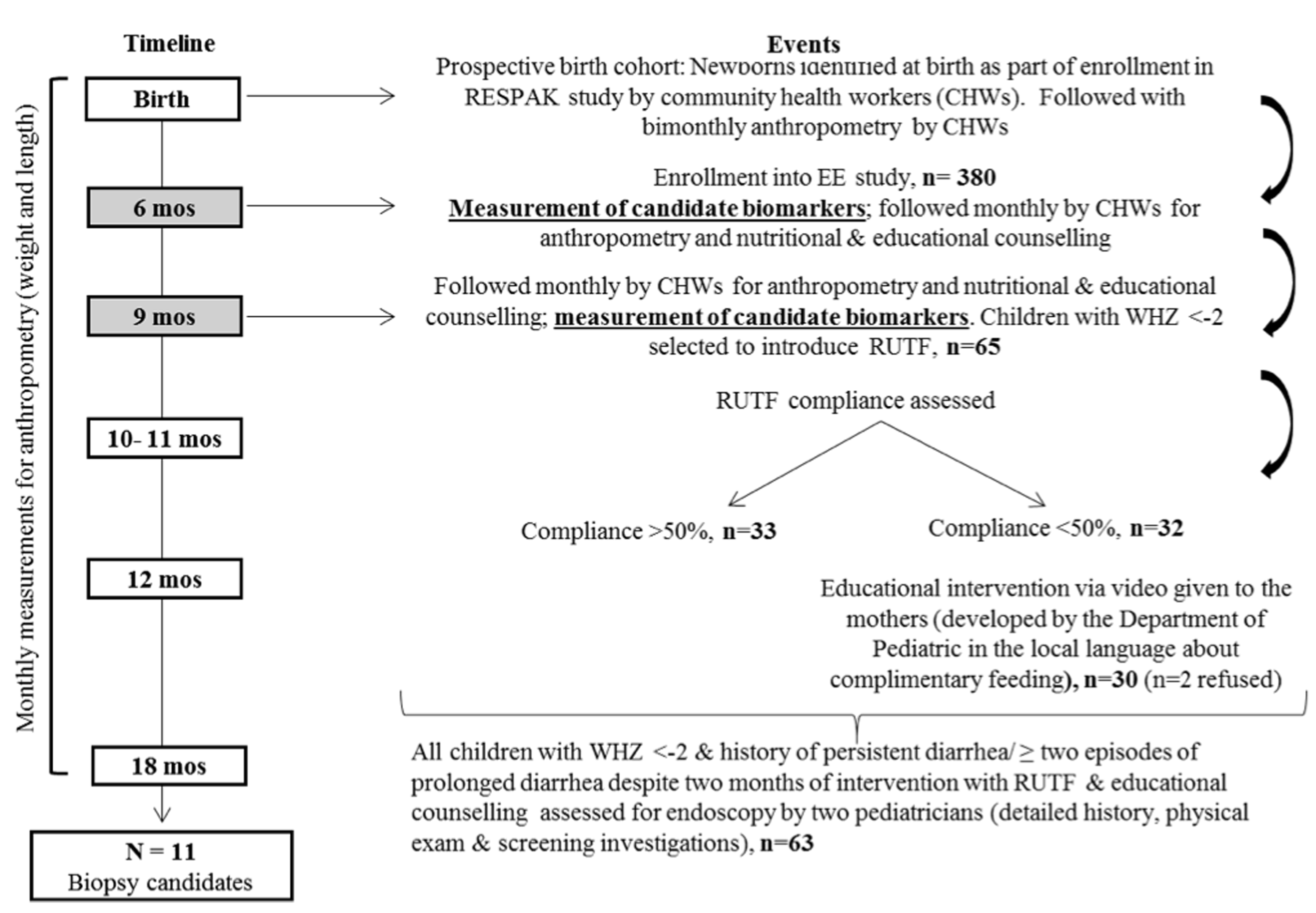

Supplement: S1 Fig — (TIF) [file pone.0193768.s001.tif]

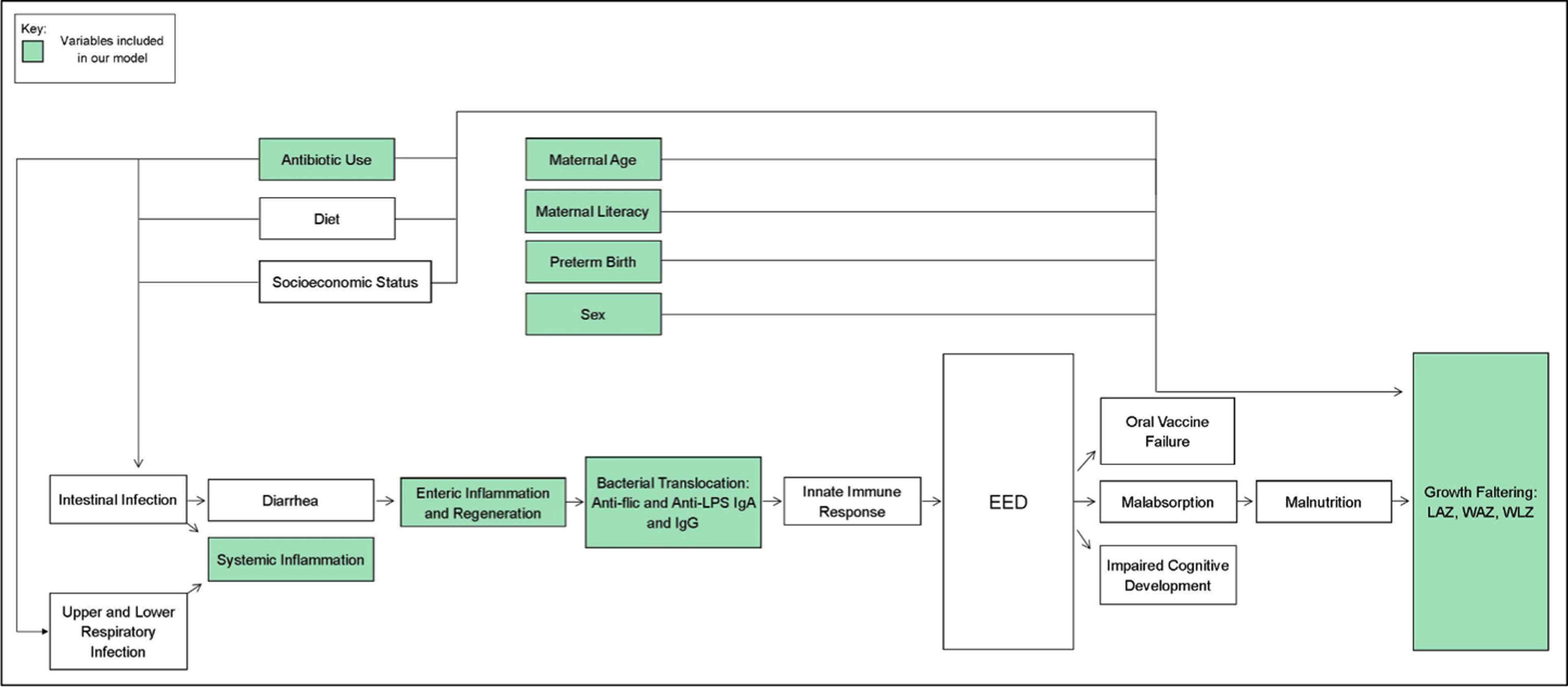

Supplement: S2 Fig — (TIF) [file pone.0193768.s002.tif]
