# Supplementary material for: Serum anti-flagellin and anti-lipopolysaccharide immunoglobulins as predictors of linear growth faltering in Pakistani infants at risk for environmental enteric dysfunction
Source: PLoS One. 2018 Mar 6;13(3):e0193768. doi: 10.1371/journal.pone.0193768 (PMC5839587; doi:10.1371/journal.pone.0193768)
Supplement: S1 Table — (DOCX) [file pone.0193768.s003.docx]

**S1 Table. Summary of Biomarkers to Assess Environmental Enteric Dysfunction from Parent Study**

| **Category** | **Biomarkers** | **Growth Analysis in current project** |
| --- | --- | --- |
| **Bacterial translocation/ Microbial translocation and immune activation** | Anti-Flic IgA, Anti-Flic IgG, Anti-LPS IgA, Anti-LPS IgG | Yes |
| **Systemic inflammation** | CRP, AGP, Ferritin | No, used for biomarker correlations only |
| **Enteric inflammation** | MPO, Neopterin |  |
| **Measures of Enteric regeneration** | Reg1b Stool, Reg1b serum |  |

Note: Abbreviations: Flic=Flagellin; LPS=Lipopolysaccharide; IgA=Immunoglobulin A; IgG=Immunoglobulin G; alpha glycoprotein=AGP; C-reactive protein=CRP; Myeloperoxidase=MPO; Neopterin=NEO; regenerating gene 1β=REG1b
