# Supplementary material for: Serum anti-flagellin and anti-lipopolysaccharide immunoglobulins as predictors of linear growth faltering in Pakistani infants at risk for environmental enteric dysfunction
Source: PLoS One. 2018 Mar 6;13(3):e0193768. doi: 10.1371/journal.pone.0193768 (PMC5839587; doi:10.1371/journal.pone.0193768)
Supplement: S2 Table — (DOCX) [file pone.0193768.s004.docx]

**S2 Table. Correlation Coefficient Matrix of Anti-Flagellin and Anti-Lipopolysaccharide Immunoglobulins**

|  | **At 6 months** | | | |  | **At 9 months** | | | | |
| --- | --- | --- | --- | --- | --- | --- | --- | --- | --- | --- |
|  | **Anti-Flic IgA, OD** | **Anti-Flic IgG, OD** | **Anti-LPS IgA, OD** | **Anti-LPS IgG, OD** |  | **Anti-Flic IgA, OD** | **Anti-Flic IgG, OD** | **Anti-LPS IgA, OD** | **Anti-LPS IgG, OD** |  |
| **At 6 months** |  |  |  |  |  |  |  |  |  |  |
| **Anti-Flic IgA, OD** |  | 0.57** | 0.63** | 0.40** |  | 0.12* |  |  |  |  |
| **Anti-Flic IgG, OD** |  |  | 0.44** | 0.64** |  |  |  |  | 0.11* |  |
| **Anti-LPS IgA, OD** |  |  |  | 0.58** |  | 0.14* |  | 0.11* |  |  |
| **Anti-LPS IgG, OD** |  |  |  |  |  |  |  |  | 0.26*** |  |
| **At 9 months** |  |  |  |  |  |  |  |  |  |  |
| **Anti-Flic IgA, OD** |  |  |  |  |  |  | 0.56** | 0.82** | 0.31** |  |
| **Anti-Flic IgG, OD** |  |  | 0.12* |  |  |  |  | 0.43** | 0.64** |  |
| **Anti-LPS IgA, OD** |  |  |  |  |  |  |  |  | 0.39** |  |
| **Anti-LPS IgG, OD** |  |  |  |  |  |  |  |  | 0.31** |  |

Note: Values are Spearman’s rank-order correlation coefficient. N = 377 for all 6 month immunoglobulins and N=324 for all 9 month immunoglobulins. ***P<0.0001; **P<0.001; *P-value<0.05. Abbreviations: Flagellin=Flic; Immunoglobulin=Ig; Lipopolysaccharide=LPS
