# Supplementary material for: Serum anti-flagellin and anti-lipopolysaccharide immunoglobulins as predictors of linear growth faltering in Pakistani infants at risk for environmental enteric dysfunction
Source: PLoS One. 2018 Mar 6;13(3):e0193768. doi: 10.1371/journal.pone.0193768 (PMC5839587; doi:10.1371/journal.pone.0193768)
Supplement: S4 Table — (DOCX) [file pone.0193768.s006.docx]

**S4 Table: The Association of Anti-Flagellin and Anti-Lipopolysaccharide Immunoglobulin Concentrations at 6 and 9 months with annual Z score changes for Weight-for-Length using Linear mixed effects models**

|  | **Immunoglobulins at 6 months with annual ΔWLZ as outcome** | | | |  | **Immunoglobulins at 9 months with annual ΔWLZ as outcome** | | | | |  |
| --- | --- | --- | --- | --- | --- | --- | --- | --- | --- | --- | --- |
|  | **Unadj β (SE)** | ***p-value*** | **Adj β (SE)**^1^ | ***p-value*** |  |  | **Unadj β (SE)** | ***p-value*** | **Adj β (SE)**^1^ | ***p-value*** |  |
| **Flic IgA, OD** |  |  |  |  |  | **Flic IgA, OD** |  |  |  |  |  |
| **Q1** <0.21 | ref | -- | ref | -- |  | **Q1** <0.32 | ref | -- | ref | -- |  |
| **Q2** 0.21 to <0.30 | 0.06(0.16) | 0.70 | 0.10(0.15) | 0.51 |  | **Q2** 0.32 to <0.44 | 0.12(0.16) | 0.46 | 0.07(0.16) | 0.66 |  |
| **Q3** 0.30 to <0.42 | 0.08(0.16) | 0.61 | 0.13(0.15) | 0.39 |  | **Q3** 0.44 to <0.61 | 0.15(0.16) | 0.33 | 0.15(0.16) | 0.35 |  |
| **Q4** >0.42 | 0.12(0.16) | 0.43 | 0.18(0.15) | 0.25 |  | **Q4** >0.61 | -0.05(0.16) | 0.76 | -0.03(0.16) | 0.86 |  |
| **Flic IgG, OD** |  |  |  |  |  | **Flic IgG, OD** |  |  |  |  |  |
| **Q1** <0.47 | Ref | -- | ref | -- |  | **Q1** <0.65 | ref | -- | ref | -- |  |
| **Q2** 0.47 to <0.60 | -0.06(0.16) | 0.68 | -0.04(0.15) | 0.80 |  | **Q2** 0.65 to <0.82 | 0.23(0.16) | 0.15 | 0.20(0.16) | 0.20 |  |
| **Q3** 0.60 to <0.75 | 0.06(0.16) | 0.72 | 0.11(0.15) | 0.48 |  | **Q3** 0.82 to <1.03 | 0.05(0.16) | 0.74 | 0.06(0.16) | 0.70 |  |
| **Q4** >0.75 | 0.17(0.16) | 0.28 | 0.24(0.15) | 0.12 |  | **Q4** >1.03 | 0.05(0.16) | 0.74 | 0.03(0.16) | 0.87 |  |
| **LPS IgA, OD** |  |  |  |  |  | **LPS IgA, OD** |  |  |  |  |  |
| **Q1** <0.17 | ref | -- | ref | -- |  | **Q1** <0.23 | ref | -- | ref | -- |  |
| **Q2** 0.17 to <0.24 | 0.38(0.15) | 0.01 | 0.34(0.15) | 0.03 |  | **Q2** 0.23 to <0.36 | 0.20(0.16) | 0.19 | 0.18(0.16) | 0.24 |  |
| **Q3** 0.24 to <0.34 | 0.17(0.16) | 0.29 | 0.19(0.15) | 0.22 |  | **Q3** 0.36 to <0.56 | -0.18(0.16) | 0.46 | -0.16(0.16) | 0.30 |  |
| **Q4** >0.34 | 0.21(0.15) | 0.18 | 0.18(0.15) | 0.24 |  | **Q4** >0.56 | 0.12(0.16) | 0.43 | 0.14(0.16) | 0.36 |  |
| **LPS IgG, OD** |  |  |  |  |  | **LPS IgG, OD** |  |  |  |  |  |
| **Q1** <0.33 | ref | **--** | ref | -- |  | **Q1** <0.44 | ref | -- | ref | -- |  |
| **Q2** 0.33 to <0.47 | -0.25(0.15) | 0.10 | -0.26(0.15) | 0.09 |  | **Q2** 0.44 to <0.62 | 0.16(0.16) | 0.30 | 0.21(0.16) | 0.19 |  |
| **Q3** 0.47 to <0.66 | 0.12(0.15) | 0.44 | 0.11(0.15) | 0.47 |  | **Q3** 0.62 to <0.89 | 0.07(0.16) | 0.65 | 0.07(0.16) | 0.64 |  |
| **Q4** >0.66 | 0.26(0.15) | 0.09 | 0.21(0.15) | 0.17 |  | **Q4** >0.89 | 0.12(0.16) | 0.45 | 0.07(0.16) | 0.66 |  |

Note: ^1^Adjusted for child sex (male/female), preterm birth (yes/no), maternal age (≥30, <30years), maternal literacy (yes/no), antibiotic use (yes/no), RUTF (yes/no). Abbreviations: Flic=Flagellin; LPS=Lipopolysaccharide; IgA=Immunoglobulin A; IgG=Immunoglobulin G; ΔLAZ= change in Length-for-age Z scores; RUTF= ready to use therapeutic food.
