# Supplementary material for: Serum anti-flagellin and anti-lipopolysaccharide immunoglobulins as predictors of linear growth faltering in Pakistani infants at risk for environmental enteric dysfunction
Source: PLoS One. 2018 Mar 6;13(3):e0193768. doi: 10.1371/journal.pone.0193768 (PMC5839587; doi:10.1371/journal.pone.0193768)
Supplement: S5 Table — (DOCX) [file pone.0193768.s007.docx]

**S5 Table. The association of Anti-Flagellin and Anti-Lipopolysaccharide Immunoglobulin Concentrations at 6 months with subsequent stunting, wasting, and underweight**

|  | **Stunting(n=127)** | | | | | **Underweight(n=145)** | | | | | **Wasting(n=235)** | | | | |
| --- | --- | --- | --- | --- | --- | --- | --- | --- | --- | --- | --- | --- | --- | --- | --- |
|  | **Events /N** | **Unadj**  **HR (95% CI)** | ***p*** | **Adj^2^**  **HR (95% CI)** | ***p*** | **Events/N** | **Unadj**  **HR (95% CI)** | ***p*** | **Adj^2^**  **HR (95% CI)** | ***p*** | **Events/N** | **Unadj**  **HR (95% CI)** | ***p*** | **Adj^2^**  **HR (95% CI)** | ***p*** |
| **Flic IgA** | | | |  |  |  |  |  |  |  |  |  |  |  |  |
| **Q1** | 20/31 | 1.00 | 0.59 | 1.00 | 0.56 | 21/36 | 1.00 | 0.94 | 1.00 | 0.95 | 28/58 | 1.00 | 0.97 | 1 | 0.81 |
| **Q2** | 30/32 | 1.05  (0.57-1.96) |  | 1.18  (0.63-2.20) |  | 18/36 | 0.76  (0.40-1.42) |  | 0.79  (0.41-1.49) |  | 19/59 | 0.62  (0.35-1.11) |  | 0.55  (0.30-1.01) |  |
| **Q3** | 20/32 | 1.05  (0.56-1.94) |  | 1.18  (0.62-2.24) |  | 19/37 | 0.84  (0.45-1.57) |  | 0.94  (0.50-1.79) |  | 25/59 | 0.84  (0.49-1.45) |  | 0.95  (0.55-1.64) |  |
| **Q4** | 16/32 | 0.83 (0.43-1.61) |  | 0.81  (0.41-1.60) |  | 19/36 | 0.94  (0.50-1.75) |  | 0.94  (0.50-1.79) |  | 24/59 | 0.89  (0.51-1.54) |  | 0.90  (0.51-1.60) |  |
| **Flic IgG** | | | | |  |  |  |  |  |  |  |  |  |  |  |
| **Q1** | 18/31 | 1.00 | 0.93 | 1.00 | 0.95 | 16/36 | 1.00 | 0.98 | 1.00 | 0.74 | 22/58 | 1.00 | 0.46 | 1.00 | 0.37 |
| **Q2** | 20/32 | 1.35  (0.71-2.55) |  | 1.43  (0.75-2.74) |  | 22/36 | 1.51  (0.79-2.88) |  | 1.62  (0.84-3.14) |  | 30/59 | 1.45  (0.84-2.51) |  | 1.44  (0.83-2.51) |  |
| **Q3** | 21/32 | 1.31  (0.70-2.46) |  | 1.33  (0.70-2.53) |  | 20/37 | 1.26  (0.65-2.43) |  | 1.29  (0.66-2.53) |  | 21/59 | 0.88  (0.48-1.60) |  | 0.74  (0.40-1.39) |  |
| **Q4** | 17/32 | 1.06  (0.54-2.05) |  | 1.06  (0.54-2.09) |  | 19/36 | 1.13  (0.58-2.19) |  | 1.27  (0.64-2.50) |  | 23/59 | 0.94  (0.53-1.70) |  | 0.91  (0.50-1.65) |  |
| **LPS IgA** | | | | |  |  |  |  |  |  |  |  |  |  |  |
| **Q1** | 18/31 | 1.00 | 0.94 | 1.00 | 0.89 | 21/36 | 1.00 | 0.84 | 1.00 | 0.79 | 25/58 | 1.00 | 1.00 | 1.00 | 0.87 |
| **Q2** | 20/32 | 1.28  (0.68-2.43) |  | **1.57**  **(0.81-3.05)** |  | 14/36 | 0.62  (0.31-1.22) |  | 0.69  (0.35-1.38) |  | 24/59 | 1.11  (0.63-1.94) |  | 1.27  (0.72-2.24) |  |
| **Q3** | 22/32 | 1.74  (0.93-3.24) |  | **2.23**  **(1.15-4.33)** |  | 26/37 | 1.43  (0.80-2.54) |  | 1.58  (0.88-2.85) |  | 23/59 | 0.91  (0.52-1.61) |  | 1.02  (0.57-1.83) |  |
| **Q4** | 16/32 | 0.96  (0.49-1.89) |  | **0.96**  **(0.48-1.91)** |  | 16/36 | 0.83  (0.43-1.59) |  | 0.86  (0.44-1.67) |  | 24/59 | 1.04  (0.60-1.83) |  | 1.13  (0.63-2.03) |  |
| **LPS IgG** | | | | |  |  |  |  |  |  |  |  |  |  |  |
| **Q1** | 19/31 | 1.00 | 0.72 | 1.00 | 0.84 | 17/36 | 1.00 | 0.67 | 1.00 | 0.31 | 28/58 | 1.00 | 0.37 | 1.00 | 0.63 |
| **Q2** | 17/32 | 0.82  (0.43-1.59) |  | 0.83  (0.42-1.64) |  | 20/36 | 1.30  (0.68-2.48) |  | 1.47  (0.76-2.84) |  | 21/59 | 0.64  (0.37-1.37) |  | 0.67  (0.37-1.20) |  |
| **Q3** | 23/32 | 1.33  (0.72-2.45) |  | 1.43  (0.76-2.69) |  | 21/37 | 1.37  (0.72-2.59) |  | 1.46  (0.76-2.81) |  | 24/59 | 0.77  (0.45-1.33) |  | 0.81  (0.46-1.43) |  |
| **Q4** | 17/32 | 0.78  (0.40-1.50) |  | 0.82  (0.42-1.59) |  | 19/36 | 1.21  (0.63-2.33) |  | 1.32  (0.68-2.57) |  | 23/59 | 0.70  (0.40-1.22) |  | 0.79  (0.45-1.39) |  |

Note: ^1^Adjusted for child sex (male/female), preterm birth (yes/no), maternal age (≥30, <30years), maternal literacy (yes/no), and antibiotic use at baseline (yes/no). Abbreviations: Flic=Flagellin; LPS=Lipopolysaccharide; IgA=Immunoglobulin A; IgG=Immunoglobulin G.
