# Supplementary material for: Serum anti-flagellin and anti-lipopolysaccharide immunoglobulins as predictors of linear growth faltering in Pakistani infants at risk for environmental enteric dysfunction
Source: PLoS One. 2018 Mar 6;13(3):e0193768. doi: 10.1371/journal.pone.0193768 (PMC5839587; doi:10.1371/journal.pone.0193768)
Supplement: S6 Table — (DOCX) [file pone.0193768.s008.docx]

**S6 Table. The Association of Continuous Anti-Flagellin and Anti-Lipopolysaccharide Immunoglobulin Concentrations at 6 and 9 months with annual Z score changes for Length using Linear mixed effects models**

|  | Biomarkers at 6 months with annual ΔLAZ as outcome | | | | Biomarkers at 9 months with annual ΔLAZ as outcome | | | |
| --- | --- | --- | --- | --- | --- | --- | --- | --- |
|  | Unadj β (SE) | p-value | Adj β (SE) | p-value | Unadj β (SE) | p-value | Adj β (SE) | p-value |
| Flic IgA, OD | -0.39 (0.27) | 0.15 | -0.39 (0.27) | 0.15 | -0.25 (0.17) | 0.14 | -0.25 (0.17) | 0.14 |
| Flic IgG, OD | -0.16 (0.19) | 0.38 | -0.16 (0.19) | 0.38 | -0.30 (0.15) | 0.05 | -0.30 (0.15) | 0.05 |
| LPS IgA, OD | -0.22 (0.19) | 0.25 | -0.22 (0.19) | 0.25 | 0.004 (0.15) | 0.98 | 0.004 (0.15) | 0.98 |
| LPS IgG, OD | -0.25 (0.16) | 0.12 | -0.25 (0.16) | 0.11 | -0.08 (0.13) | 0.53 | -0.08 (0.13) | 0.53 |

Note: β (SE) estimates no change controlling for child sex (male/female), preterm birth (yes/no), maternal age (≥30, <30years), maternal literacy (yes/no), and antibiotic use at baseline (yes/no). Abbreviations: Flic=Flagellin; LPS=Lipopolysaccharide; IgA=Immunoglobulin A; IgG=Immunoglobulin G; ΔLAZ= change in Length-for-age Z scores
